# Nrf2 and NF-κB Signaling Pathways Contribute to Porphyra-334-Mediated Inhibition of UVA-Induced Inflammation in Skin Fibroblasts

**DOI:** 10.3390/md13084721

**Published:** 2015-07-31

**Authors:** Jina Ryu, Mi-Jin Kwon, Taek-Jeong Nam

**Affiliations:** Department of Food and Life Science, Pukyong National University, Busan 608-737, Korea; E-Mails: othilia@hanmail.net (J.R.); kwonmj1108@naver.com (M.-J.K.)

**Keywords:** porphyra-334, Nrf-2, NF-κB, oxidative stress

## Abstract

In this study, we examined the protective effects of porphyra-334 against UVA-irradiated cellular damage and elucidated the underlying mechanisms. Porphyra-334 prevented UVA-induced cell death and exhibited scavenging activities against intracellular oxidative stress induced by UVA irradiation in skin fibroblasts. We found that porphyra-334 significantly reduced the secretion and expression of IL-6 and TNF-α, reduced nuclear expression of Nuclear factor-κB (NF-κB), and sustained NF-E2-related factor 2 (Nrf2) activation. Further mechanism research revealed that porphyra-334 promoted the Nrf2 signaling pathway in UVA-irradiated skin fibroblasts. Our results show that the antioxidant effect of porphyra-334 is due to the direct scavenging of oxidative stress and its inhibitory effects on NF-κB-dependent inflammatory genes, such as IL-6 and TNF-κ. Therefore, we hypothesize that boosting the Nrf2- NF-κB-dependent response to counteract environmental stress is a promising strategy for the prevention of UVA-related damage.

## 1. Introduction

Human skin is an efficient protective barrier from constant exposure to environmental stressors, such as ultraviolet (UV) radiation and harmful chemicals. Specifically, solar UV irradiation is a major environmental hazard that generates reactive oxygen species (ROS), induces DNA damage and ultimately results in skin inflammation, photoaging and cancer development [1]. During environmental stress, ROS levels increase dramatically and induce significant damage to cell structures, causing skin aging [2,3]. Additionally, the skin cells have evolved cytoprotective antioxidant defense systems and detoxifying enzymes, scavenging harmful ROS [3,4]. 

Several studies have demonstrated that NF-E2-related factor 2 (Nrf2) activation efficiently protects cells from ROS-induced damage *in vivo* and *in vitro* by inducing the expression of numerous detoxifying enzymes and antioxidant proteins. Activation of the Nrf2/ARE pathway could increase nuclear localization of Nrf2 and induce the expression of the Nrf2/ARE-dependent genes, such as *heme oxygenase-1 * (HO-1) and *NAD(P)H: quinone oxidoreductase 1* (NQO-1) [5,6].

In skin, Nrf2 activation can be triggered by UV radiation or phytochemicals in keratinocytes, fibroblasts and melanocytes *in vitro* [7,8,9]. Nrf2 activation was frequently associated with the protective effects against UVA irradiation [7,10,11]. 

Mycosporine-like amino acids (MAAs) possess significant chemoprotective effects against photo-induced skin senescence [12]. MAAs found in and isolated from a number of marine organisms, such as cyanobacteria, algae, and heterotrophic bacteria, have attracted a great deal of interest, especially for potential UV protection. A recent study suggested that MAAs have antioxidant properties and UV absorbance activities [13]. An important MAA is porphyra-334, which reportedly acts mainly in photoprotection but also possesses antioxidative abilities. Results from a recent study showed that algae extracts prevent UV-induced photodamage in human keratinocytes [9]. Previously, we extracted porphyra-334, the most abundant MAA in *Porphyra yezoensis*, which inhibited UVA-induced cellular senescence in human skin fibroblasts [14]. In this study, we evaluated the effect of porphyra-334 on UVA irradiation-treated skin fibroblasts, identified the possible mechanisms involved, and investigated Nrf2/NF-κB signaling pathways. 

## 2. Results 

### 2.1. Effect of Porphyra-334 on UVA-Induced Proinflammatory Cytokine Production

**Figure 1:**
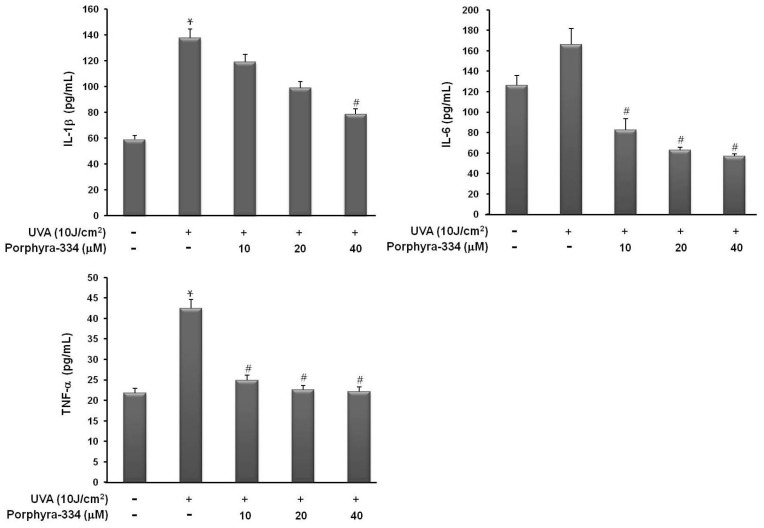
Effect of porphyra 334 on UVA-induced proinflammatory cytokine production Cells were exposed to UVA irradiation and incubated with porphyra 334 for 24 h. The concentration of cytokines in the supernatants was determined by ELISA. Values are the mean ± S.E. of triplicate experiments. * *p* < 0.05 *vs.* corresponding control, ^#^ *p* < 0.05 *vs.* corresponding only UVA irradiation.

First, to determine whether porphyra-334 protects against UVA-induced proinflammatory cytokine production in skin fibroblasts, we measured the levels of several cytokines from culture supernatants after UVA irradiation with or without porphyra-334. As shown in Figure 1, the production levels of TNF-α, IL-1β, and IL-6 in the cell supernatant were markedly increased after 10 J/cm^2^ UVA stimulation, but they were not increased during treatment with porphyra-334. Proinflammatory cytokine secretion by UVA-irradiated human skin fibroblasts was protected by porphyra-334 treatment in a dose-dependent manner. These results indicated that the presence of porphyra-334 effectively recovered the increased production of inflammatory cytokines by UVA irradiation. 

### 2.2. Effect of Porphyra-334 on UVA-Induced Inflammatory Response

**Figure 2:**
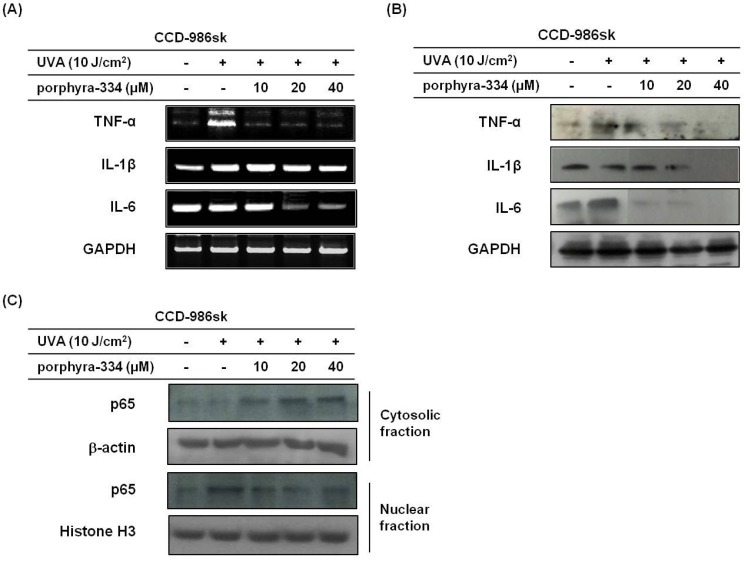
Porphyra 334 treatment can reduce inflammatory factors. (**A**) Cells were exposed to UVA irradiation and incubated with porphyra-334 for 24 h. The expression levels of cytokine genes were detected using RT-PCR. GAPDH was used as an internal standard; (**B**) The expression levels of cytokine proteins were determined by western blot analysis. GAPDH was used as an internal standard; (**C**) Some cells were fractionated into cytosolic and nuclear fractions and immunoblotted for p65, β-Actin and Histone H3.

To determine the protective effects of porphyra-334 on UVA-induced inflammation, the basal and UVA irradiation-modulated expression of TNF-α, IL-1β, and IL-6 in human skin fibroblasts were evaluated using RT-PCR and western blot analysis. As shown in Figure 2A,B, TNF-α mRNA and protein expressions were significantly increased by UVA irradiation compared with basal levels. However, treatment with porphyra-334 at a concentration of 10 μM dramatically suppressed its expression. Additionally, IL-6 mRNA and protein expression was reduced in response to porphyra-334 at a concentration of 20 μM. In accordance with the cytokine secretion, porphyra-334 was the most efficient in preventing the expression of proinflammatory cytokines TNF- and IL-6, except IL-1β, mRNA and protein levels despite cells sustaining damage from UVA irradiation. 

Because porphyra-334 suppressed the expression of proinflammatory cytokines, we evaluated the effect of porphyra-334 on the NF-κB transcription factor responsible for cytokine expression (Figure 2C). To estimate changes in the translocation of NF-κB induced by porphyra-334 in UVA-treated skin fibroblasts, western blotting was used. As expected, UVA irradiation significantly increased the expression of nuclear NF-κB p65, whereas the nuclear NF-κB p65 level was significantly reduced in porphyra-334-treated cells. These results indicated that porphyra-334 modulates NF-κB activation showing changes in nuclear NF-κB levels were caused by porphyra-334 treatment in UVA-irradiated cells. 

### 2.3. Porphyra-334 Induces the Nuclear Translocation of Nrf2 and Triggers Nrf2-Dependent Induction of HO-1 Expression Levels in Human Skin Fibroblasts

**Figure 3:**
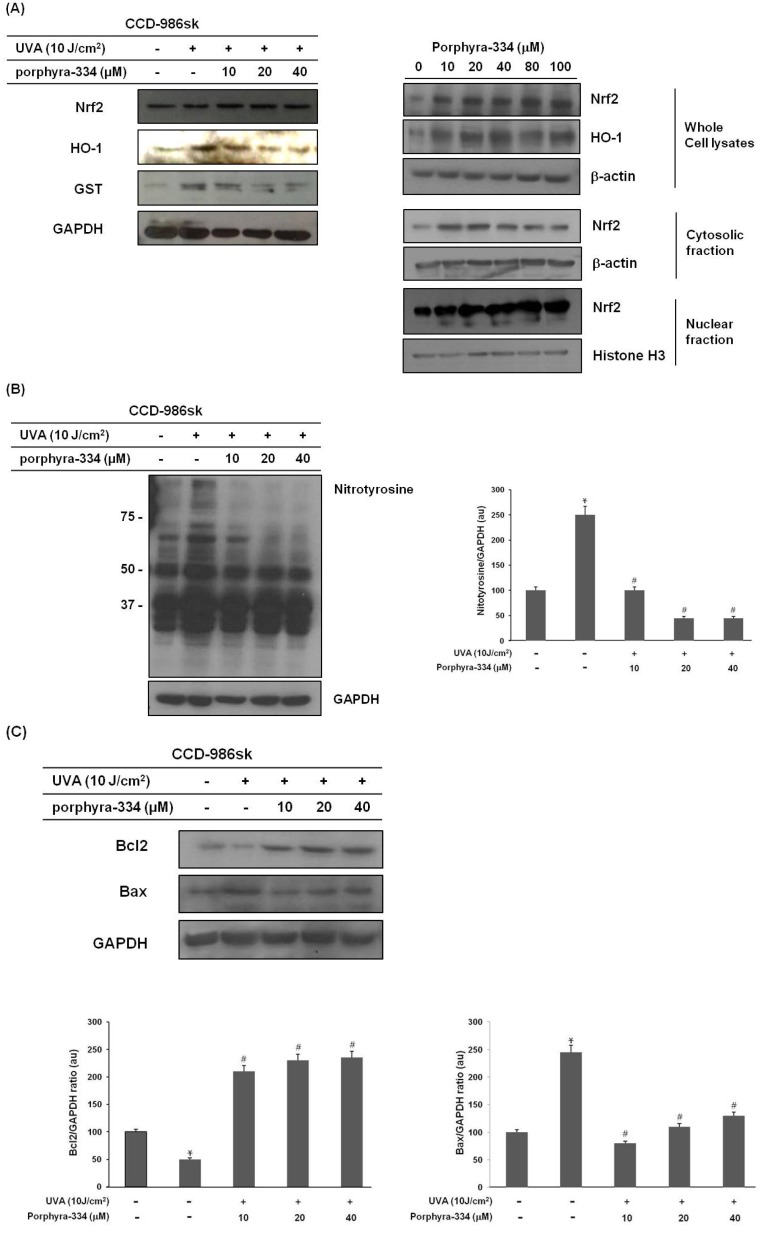
Treatment with porphyra-334 inhibited the impact of oxidative stress induced by UVA irradiation and recovered UVA-induced cell damage. (**A**) Cells were fractionated into cytosolic and nuclear fractions and immunoblotted for Nrf2, β-actin and Histone H3; (**B**) Cell lysates were immunoblotted for nitrotyrosine; (**C**) Cell lysates were immunoblotted for Bax and Bcl2. Bax/GAPDH and Bcl2/GAPDH ratio are shown below. Mean ± SE, *n* = 3. * *p* < 0.05 *vs.* corresponding control, ^#^ *p* < 0.05 *vs.* corresponding only UVA irradiation.

Important anti-inflammatory mechanisms are mediated by Nrf2 [6]. Nrf2 activation is associated with its dissociation from the Keap1-Cul3 complex that sequesters Nrf2 translocation to the nucleus and its binding to antioxidative response elements [7]. We examined the effect of porphyra-334 on the subcellular localization of Nrf2 (Figure 3A). Western blot analysis showed the Nrf2 unclear translocation was increased by porphyra-334. Additionally, porphyra-334 (10 μM) resulted in an induction of HO-1 expression. At an increased Nrf2 expression, treatment with porphyra-334 dose-dependently reduced the protein expression of HO-1 accompanying the reduction in GST levels in UVA-irradiated cells. These results suggest that porphyra-344 naturally induced Nrf2 activation and led to the recovery of UVA-induced skin fibroblast damage *via* expression of antioxidative enzymes such as HO-1 and GST, increasing the antioxidative capacity of porphyra-334-treated cells. 

### 2.4. Porphyra-334 Inhibits UVA-Induced Oxidative Stress and Prevents Cell Death

To clarify the relationship between the protective role of porphyra-334 and the change in oxidative stress, we evaluated nitrotyrosine (NT) expression, which reflects oxidative stress. As shown in Figure 3B, treating the skin fibroblast cells with UVA irradiation resulted in the upregulation of NT expression levels, especially 50–250 kDa. However, the porphyra-334 treatment decreased the NT expression levels, indicating reduced oxidative stress in porphyra-344-treated skin fibroblast cells. Our experiments showed that porphyra-334 can effectively resist UVA irradiation and remove intracellular oxidative stress. 

To determine whether porphyra-334 led to changes in Bcl2 family protein levels in UVA irradiation-treated skin fibroblasts, Bcl2 and Bax protein expressions were examined. As shown in Figure 3C, the Bax protein was maximally detected with UVA irradiation and was reduced dose-dependently in porphyra-334 treated cells. Additionally, porphyra-334 significantly increased Bcl2 protein levels as opposed to Bax protein levels. These results indicate that UVA irradiation-induced cell death is mediated by oxidative stress and that porphyra-334 exerts a potent scavenging effect, preventing UVA-induced cell death.

## 3. Discussion

Recent studies have demonstrated the therapeutic potential of porphyra-334 in photoaging [13,14]. In the current study, we showed that porphyra-334 exerted protective effects on skin cell death in UVA-damaged cells and suppressed inflammatory responses and oxidative stress, which might be associated with the regulation of Nrf2 and NF-κB activity. 

The Kelch-like ECH-associated protein 1 (Keap1)-Nrf2 system regulates the expression of cytoprotective genes in response to oxidative stresses. Nrf2 regulates the basal and inducible expression of detoxifying and antioxidant genes such as NQO1 and HO-1. 

Nrf2 includes the gene expression of antioxidant and detoxification enzymes and has been reported to exert anti-inflammatory effects by regulating several proinflammatory genes. Therefore, we believe that boosting the Nrf2-dependent response to counteract environmental stress is a promising strategy for preventing inflammation-related damage. Furthermore, Nrf2 is considered a key target of antioxidant enzyme inducers in the primary defense mechanism against ROS, converting highly toxic ROS to less reactive and less damaging forms.

HO-1 is also involved in anti-inflammation, and proinflammatory cytokines and LPS triggered HO-1 expression. However, HO-1 inhibition blocked LPS and proinflammatory cytokines-stimulated iNOS expression and NO production [15]. It is indicating that HO-1’s induction commonly occurs in the setting of increased cellular stress to help maintain physiological homeostasis and the potential for a proinflammatory environmental develops. Recently, the increased liver and adipose tissue HO-1 levels predicted unhealthy obesity in humans, and HO-1 deletion prevented metabolic disease [16]. These findings identify HO-1 inhibition as a potential therapeutic strategy for metabolic disease. In this paper, porphyra 334 inhibited UVA-induced inflammatory response, and porphyra 334 dose-dependently reduced the HO-1 protein expression in UVA-irradiated cells. Thus, we assembled HO-1 expression predicts UVA-induced skin damage, and these findings might consider HO-1 inhibition as a potential therapeutic strategy for skin damage. 

As Nrf2 is a transcription factor with potent antioxidant effects against cell death caused by ROS-induced damage, targeting Nrf2 might play an essential role in the protection against various inflammatory diseases. Because porphyra-334 activated Nrf2 in our study, it is possible that the Nrf2 triggered by porphyra-334 suppresses NF-κB activation, contributing to the anti-inflammatory effect of porphyra-334. Reportedly, Nrf2 can directly inhibit the expression of NF-κB, resulting in the reduced expression of proinflammatory cytokines caused by lipopolysaccharide (LPS) [17,18]. Although these results reveal the possibility that porphyra-334 suppresses NF-κB via Nrf2, the relationship between Nrf2 and NF-κB activity remains to be elucidated. Nevertheless, our results suggest that porphyra-334 has potent anti-inflammatory and antioxidative capabilities, which may operate by both suppressing NF-κB and activating Nrf2. 

**Figure 4:**
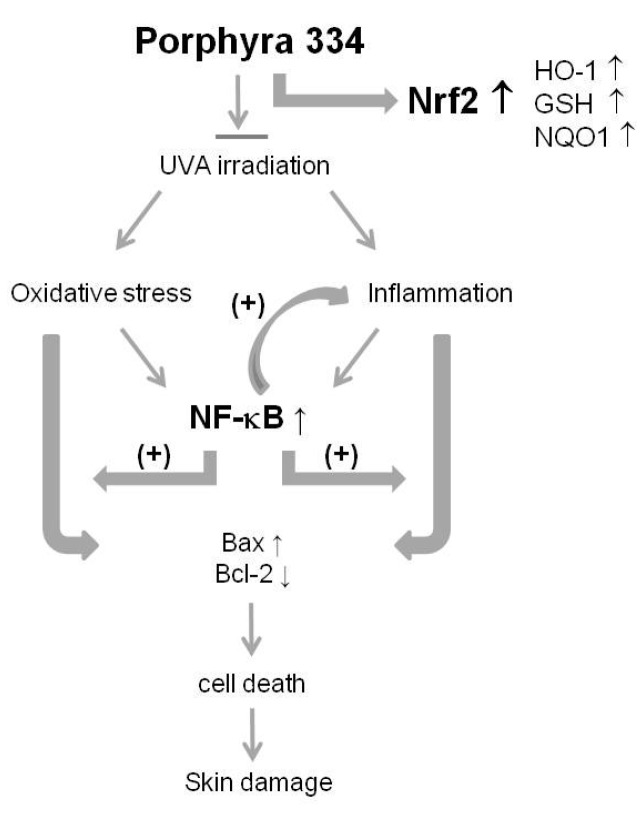
Model of porphyra-334’s role in UVA-irradiated skin fibroblasts. Boosting the Nrf2/NF-κB-dependent response to counteract environmental stress with porphyra-334 is a promising strategy for UVA-related damage prevention. The antioxidant effect of porphyra-334 is due to the direct scavenging of oxidative stress and its inhibitory effects on NF-κB-dependent inflammation genes, leading to inhibition of skin cell death.

Inflammatory cytokines have critical roles in various pathologies including skin damage [19,20]. Proinflammatory cytokines amplify the inflammatory cascade by activating inflammation-associated oxidative bursts in some cells. The crosstalk between oxidative stress and inflammation is due to the activation of NF-κB and inhibition of Nrf2 [21]. The organized regulation of Nrf2 and NF-κB has a crucial role in converting cellular signals into anti-inflammatory responses. Extensive evidence suggests that disruption or inhibition of Nrf2 signaling augments the expression and/or activity of proinflammatory regulators and sustains inflammation [21,22]. Many natural compounds have exhibited simultaneous induction of Nrf2-regulated cytoprotective protein expression and inhibition of NF-κB-regulated proinflammatory signaling [22,23]. Taken together, our results showed that porphyra-334 prevented the UVA irradiation-induced inflammation and inhibited skin cell death by regulating Nrf2 and NF-κB signaling pathways. Nrf2 and NF-κB signaling pathways may be critical to porphyra-334’s prevention of UVA damage (Figure 4). Therefore, porphyra-334 may be an attractive prevention agent for UVA-related diseases.

## 4. Experimental Section 

### 4.1. Extraction and Isolation of Water-Soluble Porphyra-334 

The porphyra-334 extraction method was performed as previously described [14]. Briefly, dried *P. yezoensis* (100 g) was extracted in hydrophilic solvent consisting of 80% aqueous methanol (v/v) at 45 °C for 2 h. The dried extract was dissolved in 150 mL ultrapure water and transferred to a separating funnel containing 666 mL chloroform-methanol-ultrapure water (2:1:1, v/v/v). The upper layer containing crude MAAs was collected. Porphyra 334 was purified using an Agilent 1100 series HPLC system equipped with a diode array detector (DAD; Agilent Technologies, Inc., Palo Alto, CA, USA). Purified porphyra 334 was stored in the dark at −70 °C until analysis. 

### 4.2. Cell Culture

Human skin fibroblasts (CCD-986sk) were obtained from the American Type Culture Collection (ATCC, Manassas, VA, USA). Cells were grown in Dulbecco’s modified Eagle’s medium (DMEM; Gibco, Grand Island, NY, USA) containing 10% (v/v) fetal bovine serum (FBS; HyClone, Logan, UT, USA) and 1% (v/v) penicillin-streptomycin (Gibco, Grand Island, NY, USA) under a humidified atmosphere of 5% CO_2_ at 37 °C.

### 4.3. UVA Irradiation and Porphyra-334 Treatment

Prior to UV irradiation, cells were washed with PBS and exposed to a 10 J/cm^2^ radiation dose of UVA light (BLX-254; Vilber Lourmat, Marne La Vallee, France) in PBS. Subsequent to irradiation, the treated cells were washed with PBS and replaced with different concentrations of porphyra-334 for 24 h. Concomitantly, no irradiation control cells were treated in the same manner, although the wells were covered with aluminum foil to prevent irradiation.

### 4.4. Reverse Transcription-Polymerase Chain Reaction (RT-PCR)

Total RNA from each sample was extracted using TRIzol reagent (Invitrogen, Carlsbad, CA, USA). According to the manufacturer’s instructions, total RNA (1 μg) was subjected to first strand cDNA synthesis using a Reverse Transceipase PreMix kit (Intron Biotechnology, Inc., Gyeonggi-do, Korea). Polymerase chain reaction (PCR) amplification of the cDNA products was performed with 2× TOPsimple™ DyeMIX (aliquot)-*n*Tag (Enzynomics, Daejeon, Korea) and primer pairs. Amplified products were separated using 1% agarose gel electrophoresis and visualized with 1 mg/mL ethidium bromide; mRNA levels were normalized using GAPDH as an internal control. The primers used in amplification are shown in Table 1.

**Table 1:** Oligonucleotide primer sequences used in RT-PCR.

Gene	Primer Sequence (5′–3′)	
	Forward Primer	Reverse Primer
TNF-α	TGCACCACAGTTTAAACCCA	GACTCCTTCAGGTGCTCAGG
IL-6	AGGAGACTTGCCTGGTGAAA	CAGGGGTGGTTATTGCATCT
IL-1β	CTGTCCTGCGTGTTGAAAGA	TTCTGCTTGAGAGGTGCTGA

### 4.5. Subcellular Fractionation 

Cytoplasmic and nuclear lysates were separated using the NE-PER extraction kit (Pierce Biotechnology, Inc., Rockford, IL, USA) according to the manufacturer’s protocol. 

### 4.6. Western Blot Analysis

After treatment, cells were washed twice with PBS, harvested, and lysed in RIPA buffer [50 mM Tris (pH 7.4), 1 mM ethylene glycol tetraacetic acid (EGTA), 150 mM NaCl, 1% Triton X-100, 0.025% sodium deoxycholate] containing protease inhibitor cocktail (Geno Technology, Inc., St. Louis, MO, USA). The lysates were centrifuged at 12,000 rpm for 15 min at 4 °C (Smart-R17; Hanil Science Industrial, Incheon, Korea). Supernatants were collected and their protein concentrations determined using a BCA protein assay kit (Pierce Biotechnology, Inc., Rockford, IL, USA). Equal amounts of protein (30 μg) were boiled for 10 min and separated using 7.5%–12% SDS-PAGE. The resolved proteins were then transferred to polyvinylidene difluoride (PVDF) membranes (Millipore Corp., Billerica, MA, USA). The membranes were blocked by incubation with 1% bovine serum albumin (BSA) in TBS-T [10 mM Tris-HCl, 150 mM NaCl (pH 7.5) containing 0.1% Tween-20] at room temperature for 1 h and incubated with specific primary antibodies (Santa Cruz Biotechnology, Inc., Dallas, TX, USA) for 3 h. The membranes were washed three times with TBS-T and incubated for 2 h with the appropriate HRP-conjugated goat anti-rabbit, goat anti-mouse, or rabbit anti-goat secondary antibody (Santa Cruz Biotechnology, Inc., Dallas, TX, USA) diluted at 1:10,000 in TBS-T 1% BSA. The respective proteins were detected with SuperSignal^®^ West Pico (Thermo Fisher Scientific, Inc., Rockford, IL, USA). The antibodies are shown in Table 2. 

**Table 2:** Primary antibodies used in western blot analysis.

Primary Antibody	COMPANY	Dilution Rate
μ-Actin	Santa Cruz Biotechnology, Inc.	1:1000
Bax	Cell Signaling TECHNOLOGY	1:1000
Bcl2	Cell Signaling TECHNOLOGY	1:1000
GAPDH	Santa Cruz Biotechnology, Inc	1:1000
HO-1	Santa Cruz Biotechnology, Inc	1:1000
IL-1β	Santa Cruz Biotechnology, Inc	1:1000
NF-κB	Cell Signaling TECHNOLOGY	1:1000
Nrf2	Enzo Life Science	1:1000
Nitrotyrosine	Cell Signaling TECHNOLOGY	1:1000
TNF-α	Santa Cruz Biotechnology, Inc.	1:1000

### 4.7. Measurement of TNF-α, IL-6 and IL-1β Production

Quantitative detection of human TNF-α, IL-6, and IL-1β production was based on standard sandwich ELISA technology using human TNF-α, IL-6, and IL-1β ELISA kits (Koma Biotech Inc., Seoul, Korea) according to the manufacturer’s instructions. 

### 4.8. Statistical Analysis

The results were presented as means ± standard error of the means (SEM) from at least three independent experiments. Data were analyzed using one-way analysis of variance (ANOVA) with Student’s *t*-test using SPSS 10.0 (SPSS, Inc., Chicago, IL, USA). A *p*-value <0.05 was considered to indicate statistical significance. 
